# MultiRNAflow: integrated analysis of temporal RNA-seq data with multiple biological conditions

**DOI:** 10.1093/bioinformatics/btae315

**Published:** 2024-05-29

**Authors:** Rodolphe Loubaton, Nicolas Champagnat, Pierre Vallois, Laurent Vallat

**Affiliations:** University of Lorraine, CNRS, Inria, IECL, F-54000 Nancy, France; University of Lorraine, CNRS, Inria, IECL, F-54000 Nancy, France; University of Lorraine, CNRS, Inria, IECL, F-54000 Nancy, France; University of Strasbourg, CNRS, UMR-7242 Biotechnology and Cell Signaling, F-67400 Illkirch, France; Department of Molecular Genetic of Cancers, Strasbourg University Hospital, F-67200 Strasbourg, France

## Abstract

**Motivation:**

The dynamic transcriptional mechanisms that govern eukaryotic cell function can now be analyzed by RNA sequencing. However, the packages currently available for the analysis of raw sequencing data do not provide automatic analysis of complex experimental designs with multiple biological conditions and multiple analysis time-points.

**Results:**

The MultiRNAflow suite combines several packages in a unified framework allowing exploratory and supervised statistical analyses of temporal data for multiple biological conditions.

**Availability and implementation:**

The R package MultiRNAflow is freely available on Bioconductor (https://bioconductor.org/packages/MultiRNAflow/), and the latest version of the source code is available on a GitHub repository (https://github.com/loubator/MultiRNAflow).

## 1 Introduction

In eukaryotic cells, genes contained in the nuclear DNA are transcribed into messenger RNA molecules before being translated into proteins that ensure physiological cellular functions. In resting cells, transcription is affected by stochastic phenomena generating a transcriptional noise within cells. After modification of the cellular environment (cellular stress, receptor activation), thousands of genes are activated, inducing a dynamic temporal transcriptional response allowing an adapted response of the cells to the initial modification of the environment (Yosef *et al.* 2013). Alterations in these temporal transcriptional responses can lead to pathologies (e.g. cancer) and are extensively studied by biologists through sometimes complex experimental designs (Bar-Joseph *et al.* 2012). Recent technological developments now make it possible to quantify the transcription of all genes in the genome by sequencing retrotranscribed RNA molecules (RNAseq).

The MultiRNAflow package is aimed at biologists and bioinformaticians who wish to automatically analyze RNAseq datasets with multiple times and/or multiple biological conditions. After unsupervised analysis of the data, the typical questions that can be addressed using our package range from selection of differentially expressed (DE) genes specific to a given biological condition or time, e.g. with the aim of inferring a gene network model specific to that biological condition, to the functional and gene ontology (GO) analyses of genes specific to a biological condition, e.g. with the aim to identify genes involved in a given cellular program (e.g. cancer cells proliferation) that are specific to a biological condition.

Several R packages propose tools to normalize data, realize unsupervised analysis and find DE genes, such as IDEAL (Marini *et al.* 2020), RNASeqR (Chao *et al.* 2021), SeqGSEA (Wang and Murray 2014), and RNAflow (Lataretu and Hölzer 2007). These packages use DESeq2 (Love *et al.* 2014) and/or EdgedR (Robinson *et al.* 2010) in order to realize the normalization and DE analysis. All of them can detect DE genes in samples belonging to different biological conditions, although RNASeqR is limited to only two biological conditions. Some of them also perform GO enrichment analyses. However, these packages were not designed to deal with temporal data, although they could be adapted to this situation. None of them offer a unified and automatized framework to analyze RNA-seq data with both several time points and more than two biological conditions. Furthermore, these packages do not allow to automatically select subsets of genes that can be relevant for GO enrichment analysis, such as genes which are specific to a given biological condition and/or to a given time, or genes with particular DE patterns.

The MultiRNAflow suite gathers in a unified framework methodological tools found in various existing packages allowing to perform: (i) exploratory (unsupervised) analysis of the data, (ii) supervised statistical analysis of dynamic transcriptional expression (DE genes), based on DESeq2 package (Love *et al.* 2014), and (iii) functional and GO analyses of genes with gProfiler2 (Kolberg *et al.* 2020) and generation of files for further analyses with several software [Webgestalt (Liao *et al.* 2019), GSEA (Subramanian *et al.* 2005)].

## 2 Supported dataset

The package supports transcriptional RNAseq raw count data (and can be adapted to single cell RNAseq) from an experimental design with multiple conditions and/or multiple times. The experimental design supported by our packages assumes that there is a reference time noted $t_{0}$, distinct from the other times noted $t_{1}$ to $t_{n}$, which corresponds to a set of reference measurements to which the others are to be compared [e.g. as in Schleiss *et al.* (2021), where $t_{0}$ is the basal state of the cell before activation of a cell receptor, and the experiments at times $t_{1}$ to $t_{n}$ measure gene expression at different times after activation of the receptor].

**Figure 1.**
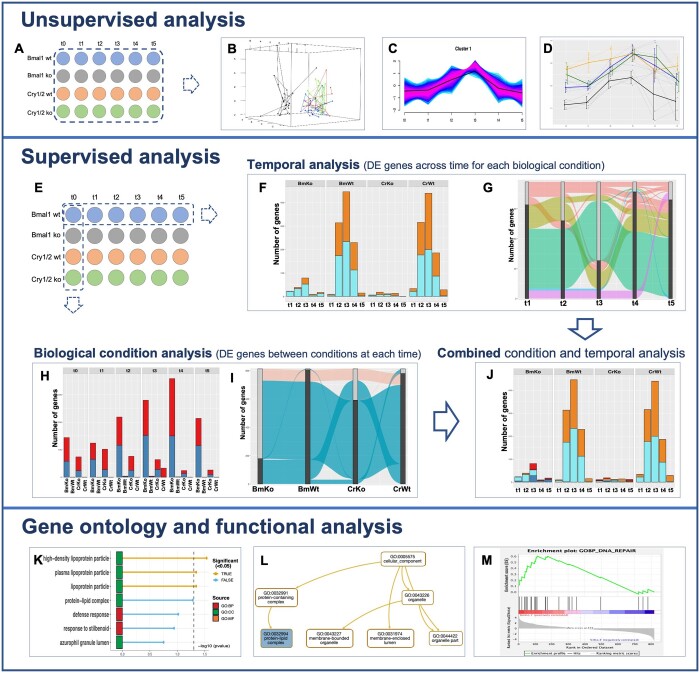
Outputs from the package MultiRNAflow with a dataset containing several biological conditions and several time points [experimental design shown in (A)]. Exploratory analysis includes 3D PCA (B), temporal clustering of expression (C), and detailed temporal gene expression (D). Supervised statistical analysis [experimental map shown in (E)] includes DE genes between each time and the reference time for each condition (F and G); specific DE genes for each condition at each time (H) or at least at one time point (I); signature DE genes of each condition and each time (J). GO enrichment analysis is realized with the R package gprofiler2 (K) or by generating input files for several GO software programs, such as Webgestalt (L) or GSEA (M)

The package provides numerous graphical outputs that can be selected by the user. To illustrate these outputs, we gather in Fig. 1 a selection of graphics obtained from the dataset of (Weger *et al.* 2021), which analyzes the role of invalidation of Bmal1 and Cry1/2 genes on murine transcriptional dynamics. The experimental map contains four biological conditions [Bmal1 wild type (wt), Bmal1 knock-out (ko), Cry1/2 wt and Cry1/2 ko] and six time points each ($t_{0}=0$ h, $t_{1}=4$ h, $t_{2}=8$ h, $t_{3}=12$ h, $t_{4}=16$ h, and $t_{5}=20$ h), with four replicates (Fig. 1A). Other outputs of the package are presented in supplemental material (Supplementary Figs S1–S11). Three other datasets with different experimental designs are presented in the package documentation (URL).

## 3 Exploratory data analysis

### 3.1 Principal component analysis and clustering

Factorial analysis of the temporal transcription of replicates for all biological conditions is performed with principal component analysis (PCA) and the visualization is optimized thanks to a dynamic 3D PCA (Fig. 1B). A hierarchical clustering on principal components (HCPC) (Lê *et al.* 2008) is also performed (Supplementary Fig. S3).

### 3.2 Hierarchical clustering based on expression and correlation

A hierarchical clustering of samples versus genes based on scaled expression data (heatmap, Supplementary Fig. S10A) allows to aggregate samples based on expression levels of each gene. A hierarchical clustering of samples versus samples based on correlations (heatmap, Supplementary Fig. S10B) allows to aggregate replicates and/or biological conditions with similar transcriptional behavior.

### 3.3 Temporal gene expression analyses

For each condition, unsupervised clustering of temporal gene expression (Fig. 1C) with Mfuzz (Kumar and Futschik 2007) highlights clusters of genes with more frequent temporal behavior within a sample. Furthermore, the graphical features of the package allow to visualize the profile of temporal expression of a gene of interest within a given cluster (Fig. 1D).

## 4 Supervised statistical analysis

From the experimental design (Fig. 1E), the supervised analysis is done either across time (DE analysis between times performed horizontally on Fig. 1E), or across biological conditions (DE analysis between conditions performed vertically on Fig. 1E), or both (combined temporal and condition DE analysis). The complete results are gathered in a csv file.

### 4.1 Temporal statistical analysis (horizontally)

For each biological condition, our package identifies DE genes ($t_{i}$ versus $t_{0}$) at each time point $t_{i}$ (Fig. 1F). In our example, with 6 time points and 4 conditions, 20 DE analyses are performed (from $t_{1}$ versus $t_{0}$ to $t_{5}$ versus $t_{0}$ for each condition). For each biological condition, an alluvial diagram (Fig. 1G) allows to follow the temporal evolution of the DE pattern of genes in each cluster of activated genes, defined as the subsets of all genes having the same first DE time (corresponding to the colors of alluvia in Fig. 1G). For each biological condition, the package also offers several graphical representations of the temporal DE analysis (Supplementary Fig. S6).

### 4.2 Biological condition statistical analysis (vertically)

For each time point $t_{i}$, we determine the DE genes between each pair of biological conditions. In our example with 6 times and 4 conditions, 24 analyses are performed. The package also identifies *specific* genes of a given condition A at time $t_{i}$, defined as the genes which are DE at time $t_{i}$ between the biological condition A and any other biological conditions, but not DE between any other pairs of biological conditions at time $t_{i}$ (Fig. 1H). In other words, these *specific* genes are the ones which, at time $t_{i}$, have a statistically different expression only in condition A compared with all other biological conditions.

For each time point, an alluvial diagram (Fig. 1I) allows to visualize the specific genes of each biological condition. The package also offers several graphical representations of the results of DE analysis between pairs of biological conditions (Supplementary Fig. S7), including volcano plots (Supplementary Fig. S9).

### 4.3 Combination of temporal and condition analyses

For each biological condition A, the combination of temporal and biological condition analyses allows to determine the set of genes that are both (i) statistically DE at least at one time in the biological condition A (and thus participating in the temporal transcriptional expression of this condition), and (ii) specific of the biological condition A at least at one time point. This set of genes (DE between $t_{i}$ and $t_{0}$ and specific for this condition at time $t_{i}$) constitutes the temporal transcriptional *signature* of the biological condition A. These genes are the most relevant of the specificity of the temporal transcriptional expression of biological condition A (represented in red and dark blue, among temporal DE genes in orange and light blue in Fig. 1J).

## 5 Functional and GO analyses

For all the lists of genes resulting from the above analyses (temporal DE genes, condition *specific* DE genes, *signature* DE genes) MultiRNAflow performs GO enrichment with the Rpackage gprofiler2 (Kolberg *et al.* 2020) (Fig. 1K). It also automatically generates outputs that can be implemented in either DAVID (Sherman *et al.* 2022), Webgestalt (Liao *et al.* 2019) (Fig. 1L), g:Profiler (Raudvere *et al.* 2019), or GSEA (Subramanian *et al.* 2005) (Fig. 1M) for further analyses.

## Supplementary Material

btae315_Supplementary_Data
