# Supplementary material for: MultiRNAflow: integrated analysis of temporal RNA-seq data with multiple biological conditions
Source: Bioinformatics. 2024 May 29;40(5):btae315. doi: 10.1093/bioinformatics/btae315 (PMC11139518; doi:10.1093/bioinformatics/btae315)
Supplement: btae315_Supplementary_Data [file btae315_supplementary_data.pdf]

Supplementary Material

MultiRNAflow :
integrated analysis of temporal RNA-seq data with multiple biological conditions

Rodolphe Loubaton, Nicolas Champagnat, Pierre Vallois and Laurent Vallat

Contents

1 Introduction **2**

2 Exploratory data analysis **2**
2.1 Normalization 2
2.2 Principal Component Analysis (PCA) and clustering 3
2.3 Temporal gene expression analyses 5
2.4 Temporal expression pattern of each gene 6

3 Supervised statistical analysis of the transcriptional response **6**
3.1 Supervised statistical analysis (differential expression (DE)) 6
3.1.1 Temporal statistical analysis 7
3.1.2 Biological condition statistical analysis 8
3.1.3 Combination of temporal (horizontally) and biological condition (vertically) statistical analyses 9
3.2 Volcano plots, ratio intensity (MA) plots and heatmaps 10

4 Functional and Gene ontology (GO) analyses **11**

1 Introduction

In this supplementary material, we illustrate all the output graphs of the R package **MultiRNAflow** with the dataset associated to the article of Weger *et al.* (2021).

2 Exploratory data analysis

2.1 Normalization

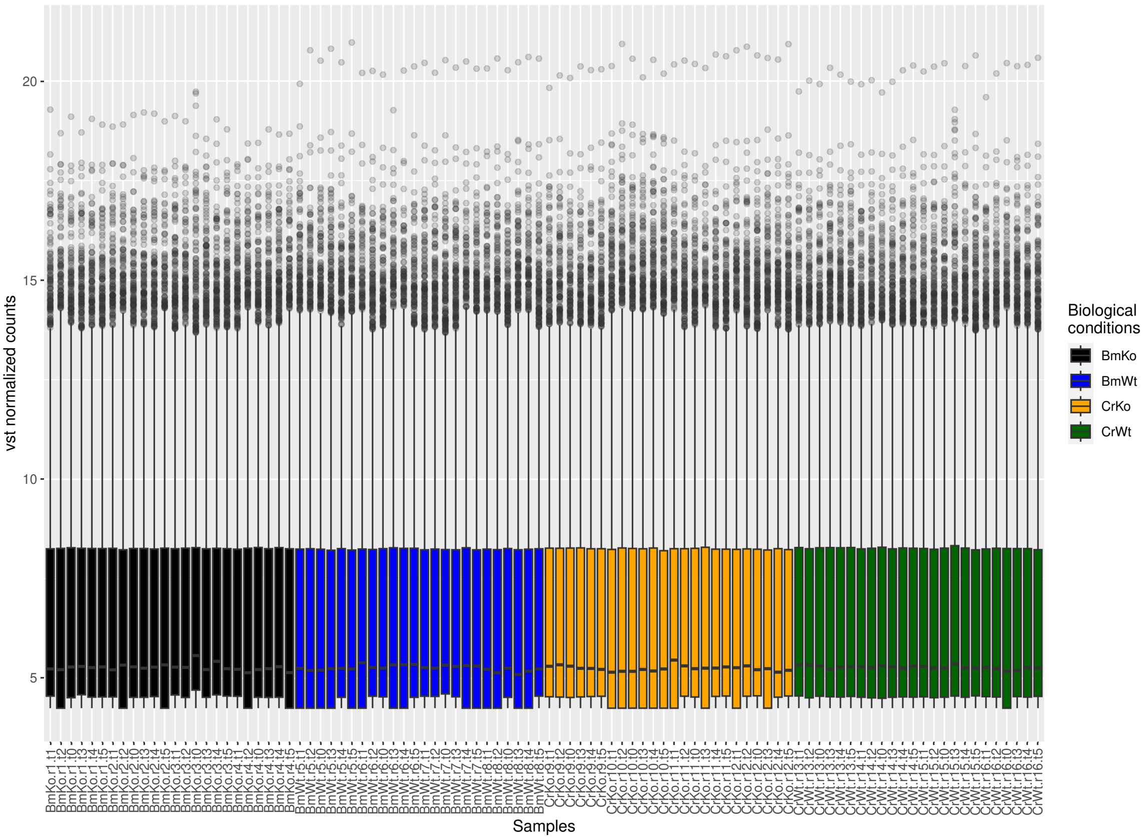

Figure S1: The function *DATAnormalization()* of **MultiRNAflow** returns a boxplot showing the distribution of the normalized expression (here using the vst method) of genes for each sample. The color of the boxplots is different for different biological conditions. The x-labels give biological conditions, time and replicate numbers separated by dots of each sample.

The function *DATAnormalization()* uses the vst method (Anders and Huber, 2010) from the R package DESeq2 (Love *et al.*, 2014).

2.2 Principal Component Analysis (PCA) and clustering

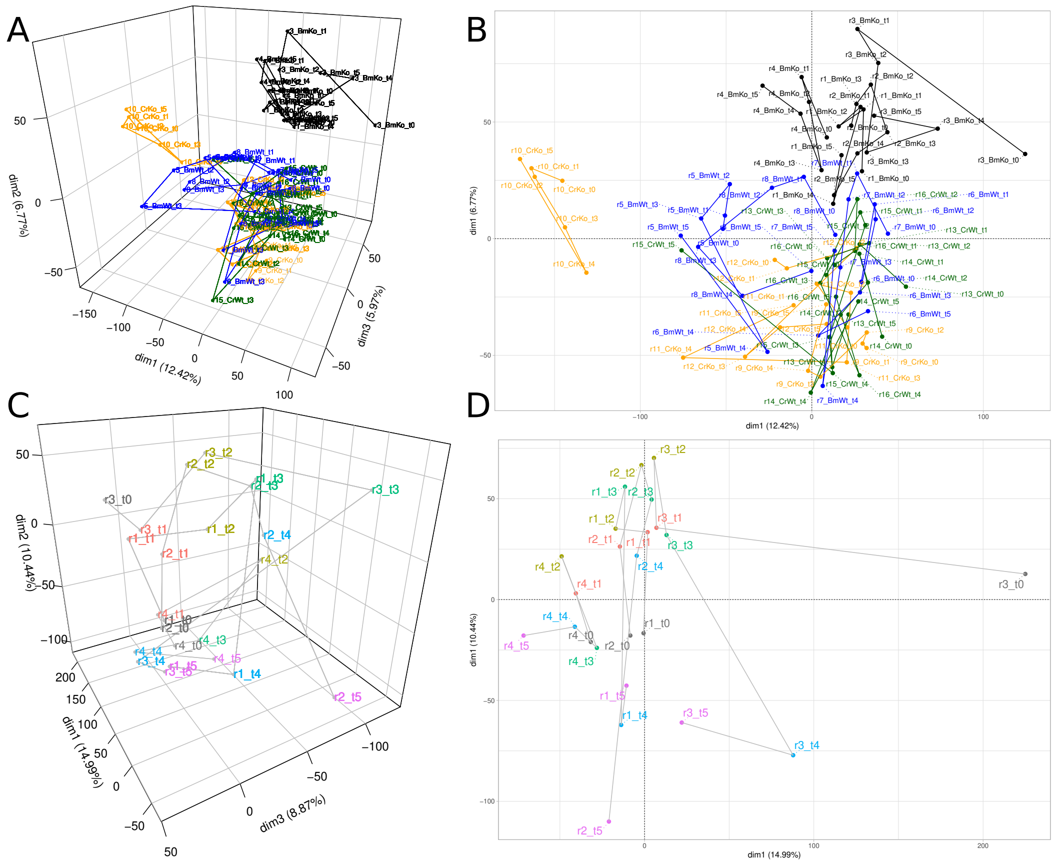

Figure S2: The PCA is realized with the R function *PCAanalysis()* of **MultiRNAflow**. When the experimental design contains several time points and several biological conditions, the function returns : one 3D PCA graph (A) and one 2D PCA graph (B) where samples are colored with different colors for different biological conditions. Furthermore, lines are drawn between each pair of consecutive points for each sample. The function also returns one 3D PCA graph (C) and one 2D PCA graph (D) for each biological condition (here BmKo only), where samples are colored with different colors for different time points. Furthermore, lines are drawn between each pair of consecutive points for each sample.

The *PCAanalysis()* function also returns the same graphs describe above but without lines. An option also allows to plot all previous 3D PCA graphs in a rgl window allowing to interactively rotate and zoom.
The function *PCAanalysis()* uses the R package FactoMineR (Lê *et al.*, 2008).

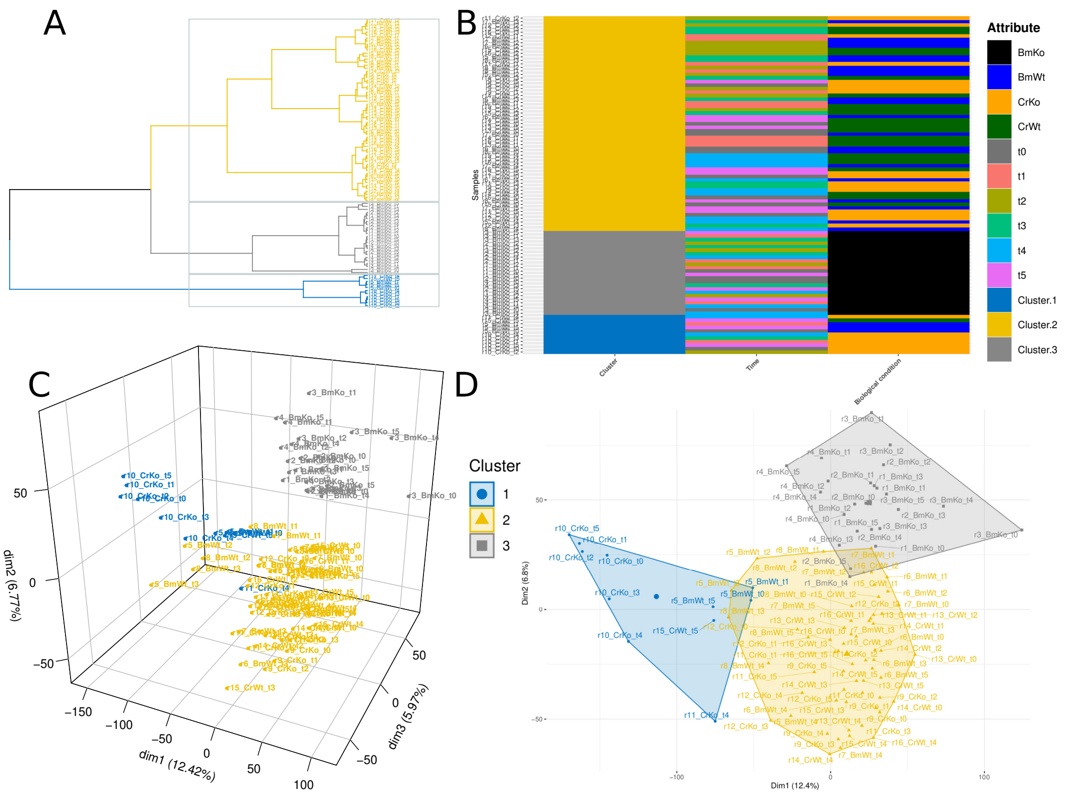

Figure S3: The R function *HCPCanalysis()* of **MultiRNAflow** performs a hierarchical clustering on principal components (HCPC). The function returns: a dendrogram (A); a graph (B) showing for each sample, its cluster (fisrt column), the associated time points (second column) and biological condition (third column) using a color code; one 3D PCA graph (C); one 2D PCA graph (D) where colors correspond to HCPC clusters.

The *HCPCanalysis()* function also returns the same 3D PCA graph as in Figure S3.C in a rgl window allowing to interactively rotate and zoom.
The function *HCPCanalysis()* uses the R package FactoMineR (Lê *et al.*, 2008).

2.3 Temporal gene expression analyses

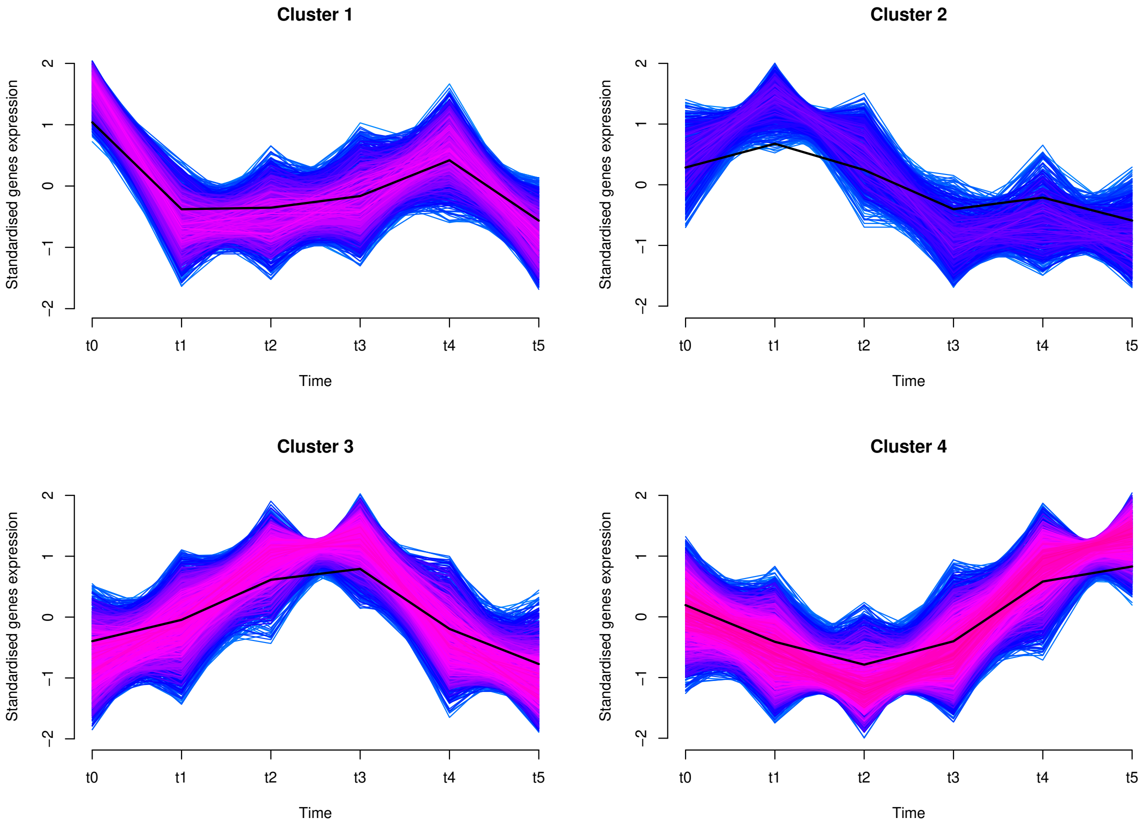

Figure S4: Soft clustering of genes is realized with the R function *MFUZZanalysis()* of **MultiRNAflow** in order to detect the most common temporal behavior among all genes for each biological condition. The figure corresponds to the results of the soft clustering for the biological condition BmKo.

The function *MFUZZanalysis()* uses the R package Mfuzz (Kumar and Futschik, 2007).

2.4 Temporal expression pattern of each gene

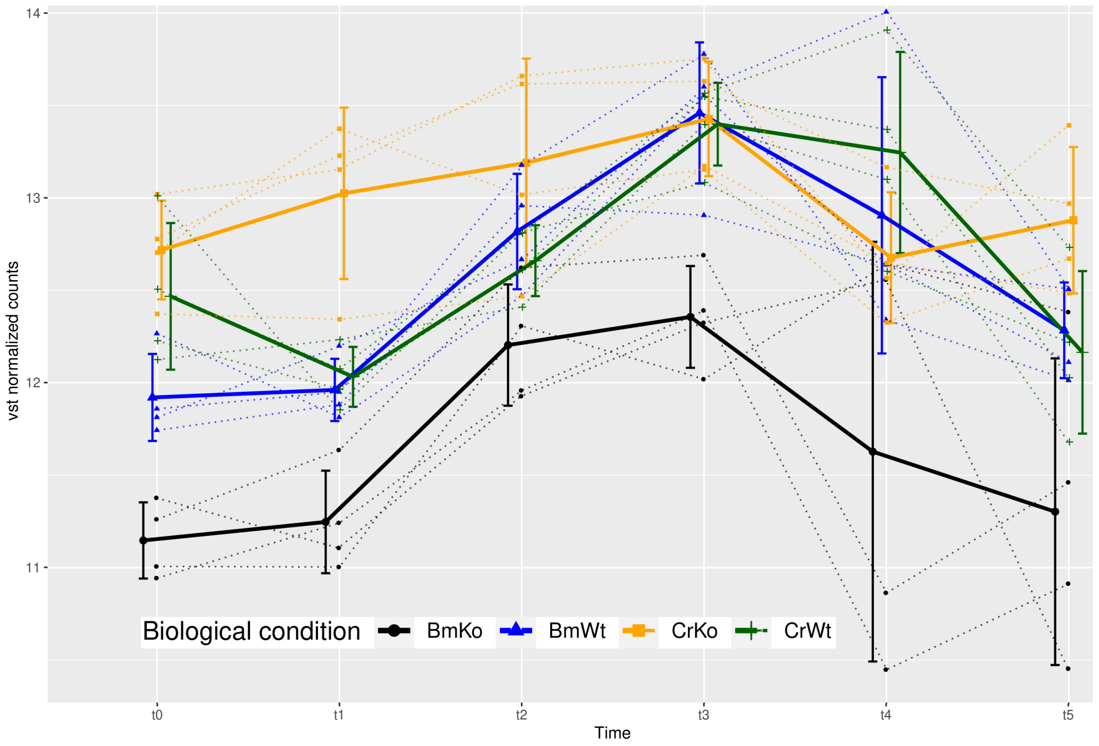

Figure S5: The function *DATAplotExpressionGenes()* of **MultiRNAflow** allows to plot for each biological condition: the temporal evolution of the four replicates of the expression of a selection of genes, and the corresponding mean and standard deviation. Colors are different for different biological conditions. Here, the temporal expression pattern of gene ENSMUSG00000031770 is shown and that gene belongs to the cluster 3 of the results of the soft clustering for the biological condition BmKo.

3 Supervised statistical analysis of the transcriptional response

3.1 Supervised statistical analysis (differential expression (DE))

The function *DEanalysisGlobal()* of **MultiRNAflow** realizes the supervised statistical analysis and uses the R package DESeq2 (Love *et al.*, 2014).

3.1.1 Temporal statistical analysis

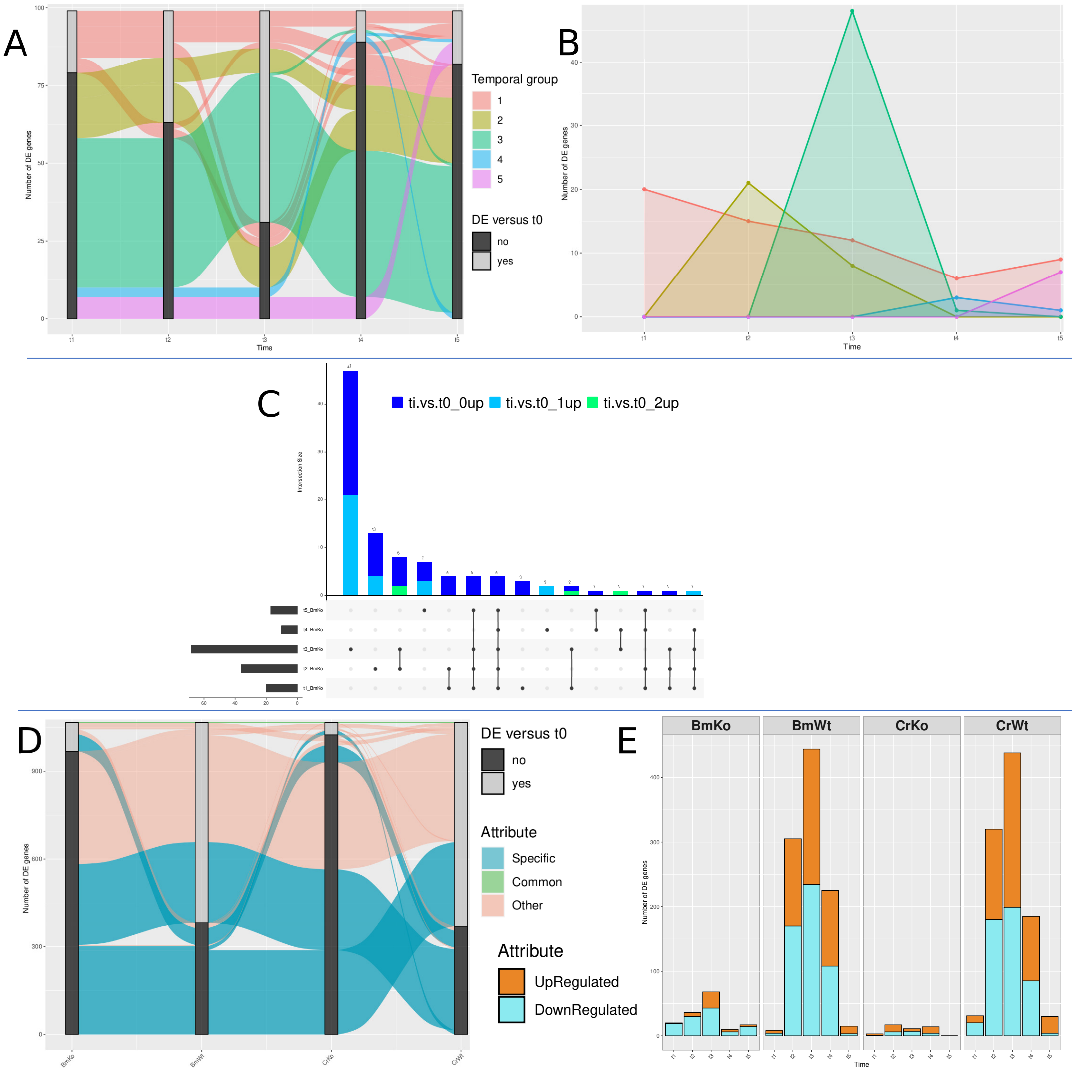

Figure S6: For each biological condition, the function returns: an alluvial diagram (A) of DE genes at least at one time, a graph (B) showing the number of DE genes as a function of time for each temporal group, a Venn barplot (C) showing the number of DE genes belonging to each DE temporal pattern. By temporal group, we mean the sets of genes which are first DE at the same time. By temporal pattern, we mean the set of times t_i such that the gene is DE between t_i and the reference time t_0. The function *DEanalysisGlobal()* also returns: an alluvial diagram (D) for DE genes at least at one time for each biological condition and a barplot (E) showing the number of DE genes up-regulated and down-regulated for each time and biological condition.

3.1.2 Biological condition statistical analysis

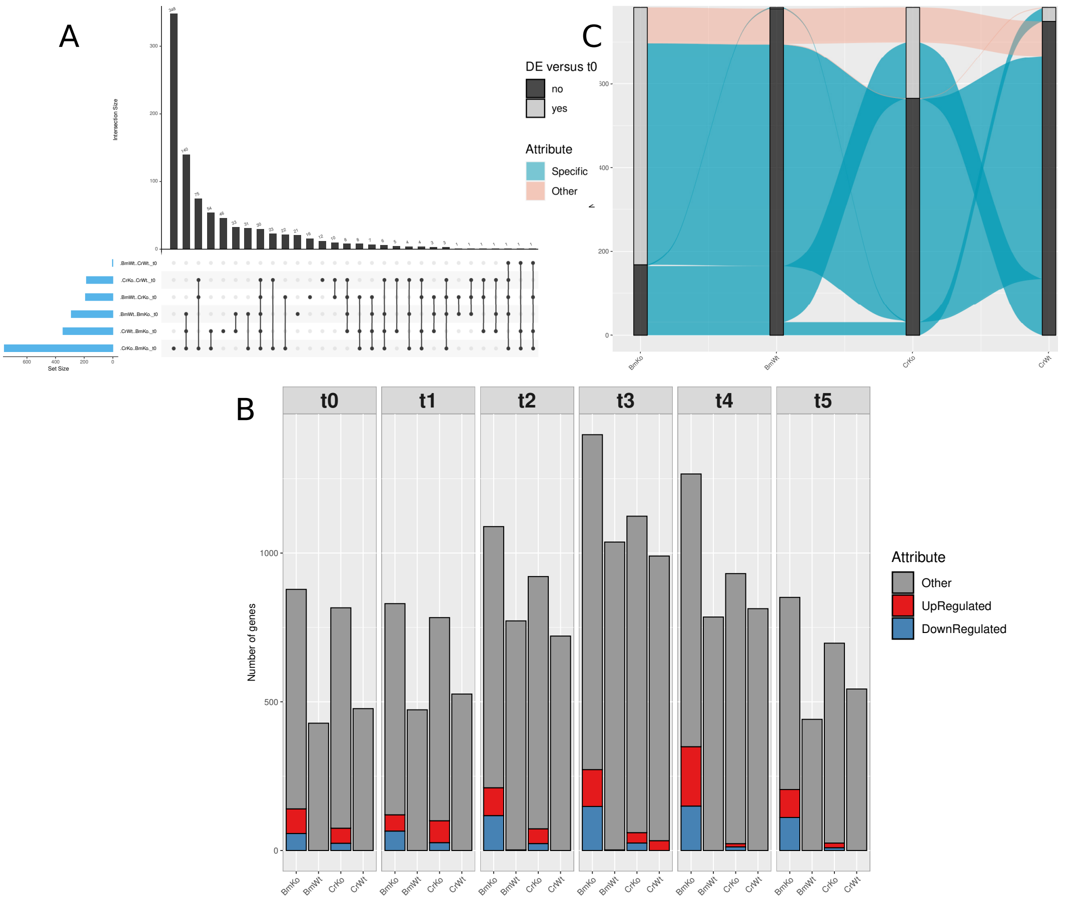

Figure S7: For each time point, the function *DEanalysisGlobal()* returns a Venn barplot (A) which gives the number of genes for each possible intersection. We say that a set of pairs of biological conditions forms an intersection if there is at least one gene which is DE for each of these pairs of biological conditions, but not for the others. The function also returns a barplot (B) showing the number of specific genes per biological condition for each time and an alluvial diagram (C) of genes which are specific at least at one time for each biological condition. A gene is called specific of a given biological condition at a time t_i, if the gene is DE between this condition and any other conditions at time t_i, but not DE between any pair of other biological conditions at time t_i.

3.1.3 Combination of temporal (horizontally) and biological condition (vertically) statistical analyses

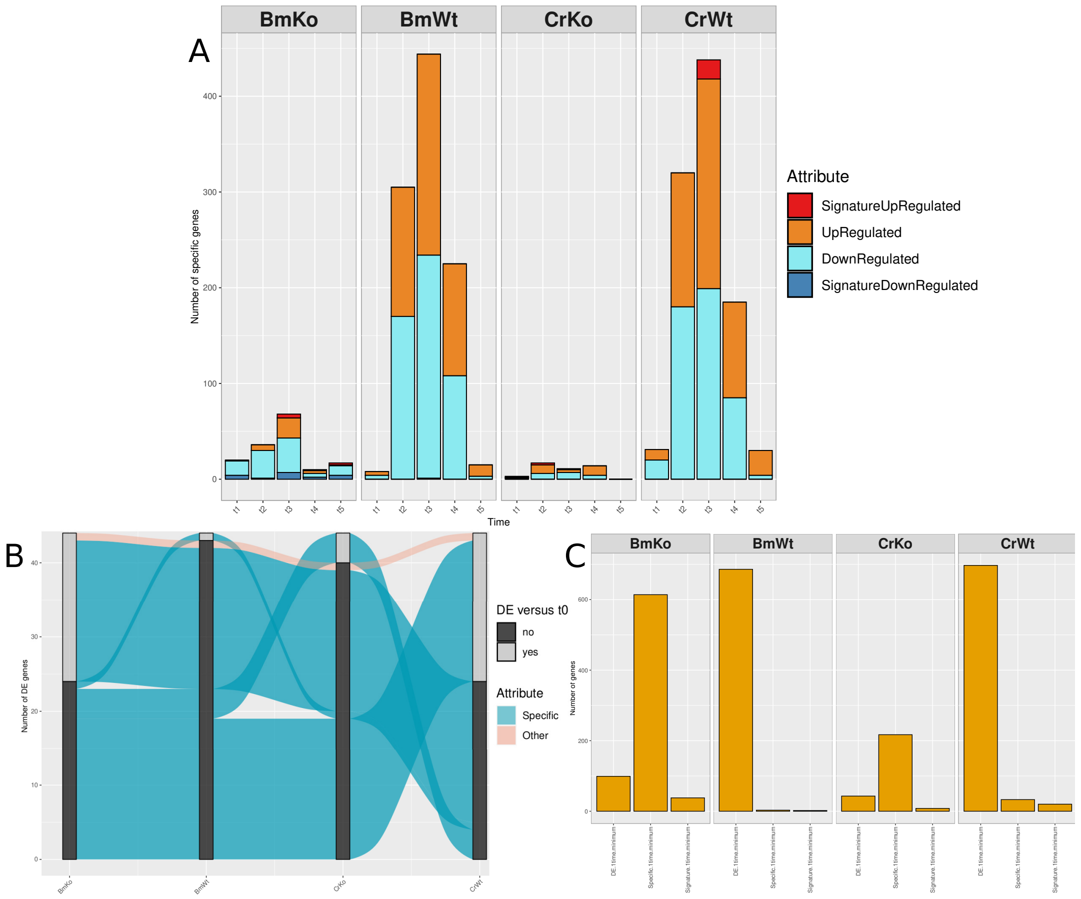

Figure S8: The function *DEanalysisGlobal()* also returns plots combining the temporal and biological condition analyses: a barplot (A) showing the number of DE genes and signature genes for each time and biological condition and an alluvial graph (B) for DE genes wich are signature at least at one time for each biological condition. A gene is called signature of a given biological condition at a time t_i, if the gene is both i) statistically DE at time t_i and thus participating in the temporal transcriptional response of this biological condition, and ii) specific of this biological condition at time t_i. This set of genes (DE between t_i and t_0 and specific for this biological condition at time t_i) constitutes the transcriptional signature of this biological condition. A barplot (C) showing the number of genes which are DE at least at one time, specific at least at one time and signature at least at one time for each biological condition

3.2 Volcano plots, ratio intensity (MA) plots and heatmaps

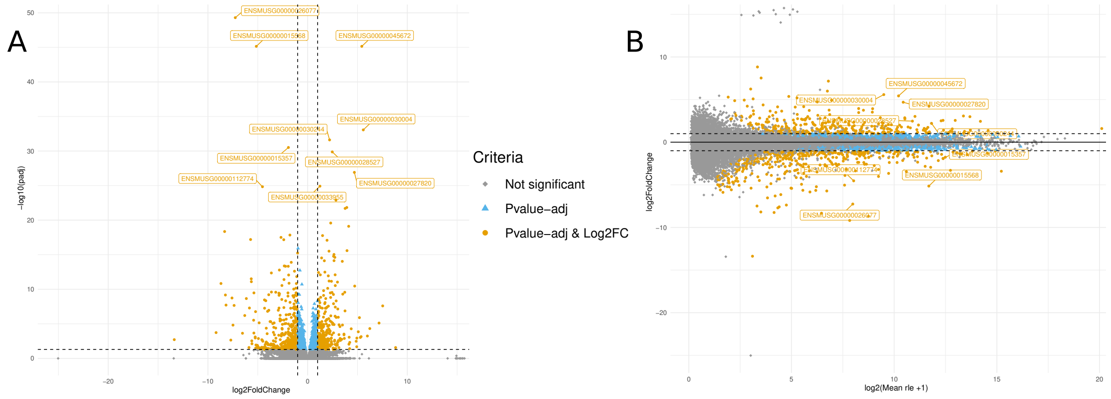

Figure S9: The function *DEplotVolcanoMA()* of **MultiRNAflow** returns volcano and MA plots for all biological conditions between each pairs of times (t_i versus t_0) and for all times between each pairs of biological condition. Figure (A) and (B) show the volcano and MA plots corresponding to BmWt versus BmKo at time t_4.

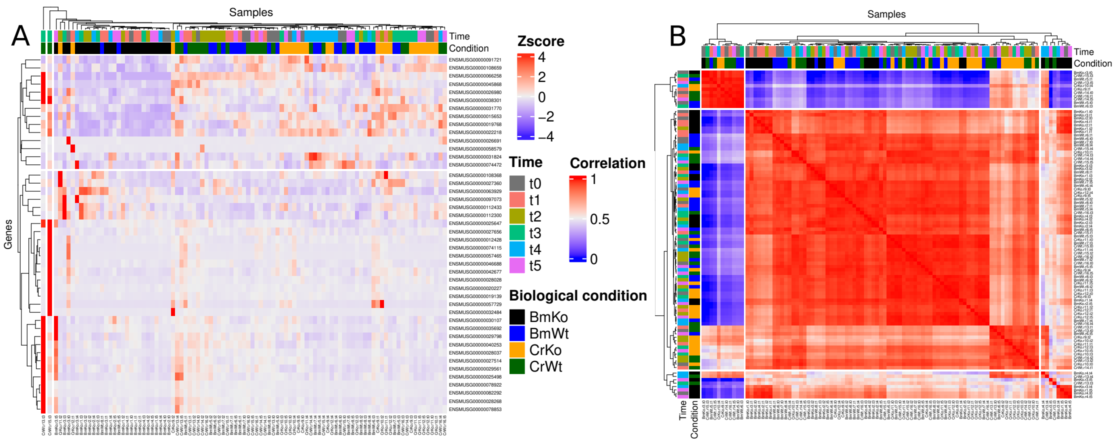

Figure S10: The function *DEplotHeatmaps()* of **MultiRNAflow** plots a heatmap based on scaled expression data (A) accross samples and genes from a subset of genes that can be selected by the user and a correlation heatmap between samples (B). Here, the genes considered are genes which are signature at least one time for the biological condition BmKo.

4 Functional and Gene ontology (GO) analyses

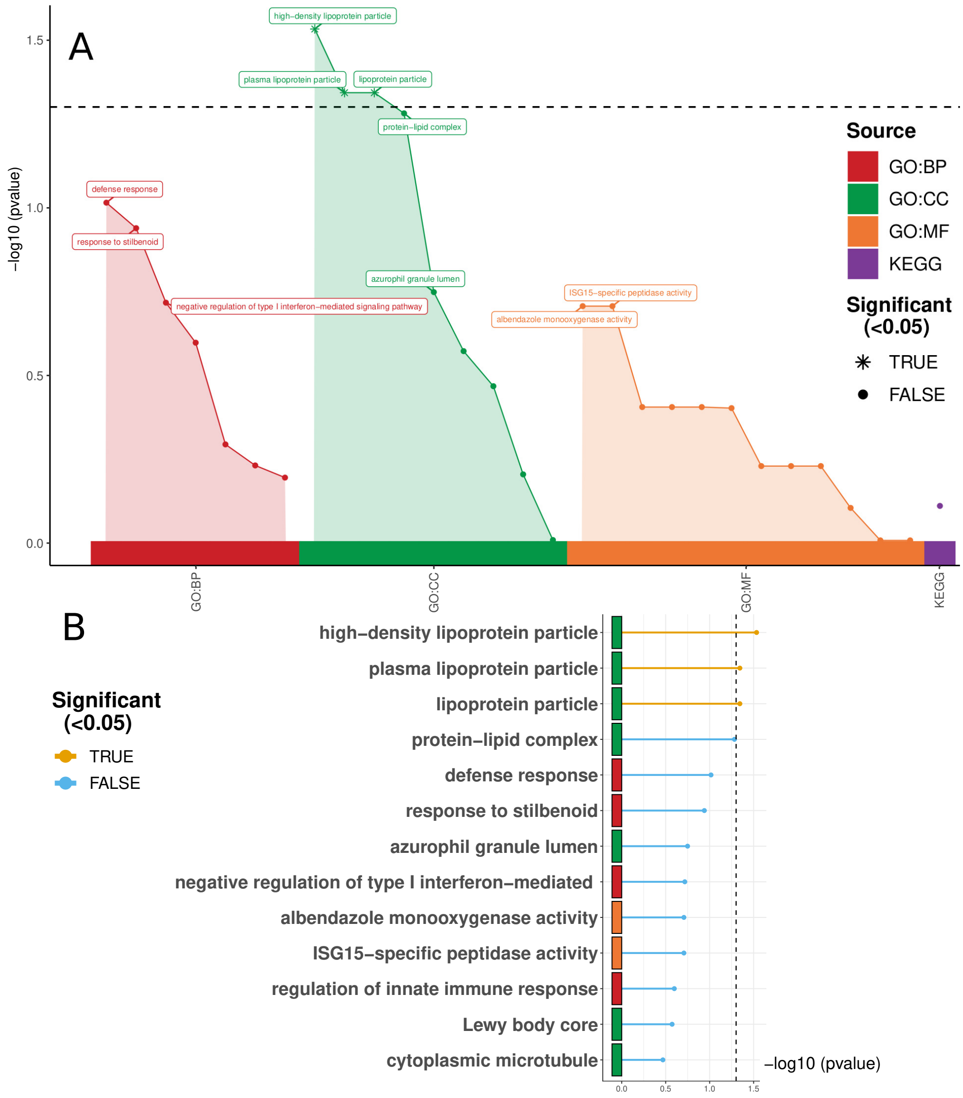

Figure S11: The function *GSEAQuickAnalysis()* of **MultiRNAflow** returns a Manhattan plot (A) indicating all genes ontologies ordered according to p-value and grouped according to the functional database (Biological Process (GO::BP), cellular component (GO::CC), Molecular Function (GO::MF) and Kyoto Encyclopedia of Genes and Genomes (KEGG)). The function also plots a lollipop graph (B) where the gene ontologies and pathways are sorted in descending order of significance. A lollipop is yellow if the p-value is smaller than 0.05 (significant) and blue otherwise.

The function *GSEAQuickAnalysis()* uses the R package gprofiler2 (Kolberg *et al.*, 2020).

References

Anders, S., Huber, W. (2010), Differential expression analysis for sequence count data, *Genome Biol*, **11**, R106.

Kolberg, L. *et al.* (2020), gprofiler2 - an R package for gene list functional enrichment analysis and namespace conversion toolset g:Profiler, *F1000Res.*, **9**, 709.

Kumar, L. , Futschik, M. (2007), Mfuzz: a software package for soft clustering of microarray data, *Bioinformation*, **2**, 5-7.

Lê, S. *et al.* (2008), FactoMineR: An R Package for Multivariate Analysis, *J. Stat. Soft.*, **25**.

Love, M.I. *et al.* (2014), Moderated estimation of fold change and dispersion for RNA-seq data with DESeq2, *Genome Biol.*, **15**, 550.

Weger, BD. *et al.* (2021), Systematic analysis of differential rhythmic liver gene expression mediated by the circadian clock and feeding rhythms, *Proc. Natl. Acad. Sci. U.S.A.*, **118**, e2015803118.
